# Full-Length Genome Characterization of Chinese Porcine Deltacoronavirus Strain CH/SXD1/2015

**DOI:** 10.1128/genomeA.01284-15

**Published:** 2015-10-29

**Authors:** Fangzhou Chen, Yinxing Zhu, Meizhou Wu, Xugang Ku, Li Yao, Qigai He

**Affiliations:** State Key Laboratory of Agricultural Microbiology, College of Veterinary Medicine, Huazhong Agricultural University, Wuhan, China

## Abstract

A porcine deltacoronavirus (PDCoV) was identified in the Chinese mainland and found to be closely related to Hong Kong strain HKU15-155 but differed from PDCoV strains in the United States and South Korea. The complete genome of PDCoV strain CH/SXD1/201 was sequenced and analyzed to further characterize PDCoV in Chinese swine.

## GENOME ANNOUNCEMENT

Porcine deltacoronavirus (PDCoV) is an enveloped, single-stranded, positive-sense RNA virus that belongs to the family *Coronaviridae*, genus *Deltacoronavirus*, with a genome that is about 25.4 kb in length (1). The disease caused by this virus is characterized by dehydration, watery diarrhea, and low mortality in adult pigs and high mortality in piglets. The clinical symptoms of PDCoV disease are very similar to those of porcine epidemic diarrhea (2), but PDCoV disease is milder (3). The PDCoV pathogen was first identified through genomic sequence analysis of avian and pig isolates in Hong Kong in 2012 (1). Since February 2014, disease caused by HKU15-44-like PDCoV strains has been reported in the United States and South Korea (4–8).

Since August 2014, 64 fecal and intestinal samples have been collected from six pig farms from Shanxi province, Guangdong province, and Hubei province in China, from which PDCoV was detected by reverse transcription (RT)-PCR, and the other general enteropathogens, such as porcine epidemic diarrhea virus, porcine transmissible gastroenteritis virus, and porcine rotavirus, were excluded by the RT-PCR method (9). The prevalence of PDCoV infection was 23.4% (15/64; 95% CI, 13.8% to 35.7%) in these samples. A PDCoV strain, CH/SXD1/2015, was detected from one piglet with severe diarrhea. To better understand the genetic characteristics of PDCoV in mainland China and the genetic relationship with other PDCoV strains reported in United States, South Korea, and Hong Kong, the complete genome of a representative strain, CH/SXD1/2015, was sequenced using the previously described method (4).

The genome of CH/SXD1/2015 was identified as 25,419 nucleotides (nt) in length excluding the 3′ poly(A) tail, three nucleotides shorter than the other U.S. and South Korean strains deposited in GenBank. The genomic structure of PDCoV, except the 3′ pol(A) tail, was 5′-UTR-ORF1-S-E-M-NSP6-N-NSP7-3′UTR, and the nucleotides numbers of these parts were 539, 18,803, 3,480, 252, 654, 285, 1,029, 603, and 392, respectively.

The complete genome sequence of CH/SXD1/2015 has 98.7% to 99.3% and 98.8% to 98.9% nucleotide identities with 7 Chinese PDCoV strains (accession numbers KP757890 to KP757892, KT266822, KR131621, JQ065042, and JQ065043) and 10 U.S. and South Korean PDCoV strains (accession numbers KM820765, KJ769231, KJ584356, KP981395, KJ567050, KJ481931, KJ601777, KJ601778, KJ601779, and KJ601780), respectively. CH/SXD1/2015 and another five recently isolated Chinese strains (accession numbers KP757891, KP757892, KT266822, KR131621, and JQ065043), except the two strains CHN-AH-2004 (accession number KP757890, isolated in 2004) and HKU15-44 (accession number JQ065042, isolated in 2009), had an amino acid deletion in amino acid 52 of the S protein.

These data will help us better understand the genetic diversity of PDCoV in China. Compared with all strains in the United States and South Korea, this unique feature, the three-nucleotide deletion in the S gene, can be used as a genetic marker to discriminate PDCoV strains in China from those in the United States and South Korea.

### Nucleotide sequence accession number. 

The full-length genome sequence of the PDCoV strain CH/SDX1/2015 has been deposited at GenBank with the accession number KT021234.
